# Analysis of high-throughput RNAi screening data in identifying genes mediating sensitivity to chemotherapeutic drugs: statistical approaches and perspectives

**DOI:** 10.1186/1471-2164-13-S8-S3

**Published:** 2012-12-17

**Authors:** Fei Ye, Joshua A Bauer, Jennifer A Pietenpol, Yu Shyr

**Affiliations:** 1 571 PRB, 2220 Pierce Avenue, Department of Biostatistics, Vanderbilt University School of Medicine, Nashville, TN 37232-6848, USA; 2 652 PRB, 2220 Pierce Avenue, Departments of Biochemistry, Vanderbilt University School of Medicine, Nashville, TN 37232-6848, USA

## Abstract

**Background:**

High-throughput RNA interference (RNAi) screens have been used to find genes that, when silenced, result in sensitivity to certain chemotherapy drugs. Researchers therefore can further identify drug-sensitive targets and novel drug combinations that sensitize cancer cells to chemotherapeutic drugs. Considerable uncertainty exists about the efficiency and accuracy of statistical approaches used for RNAi hit selection in drug sensitivity studies. Researchers require statistical methods suitable for analyzing high-throughput RNAi screening data that will reduce false-positive and false-negative rates.

**Results:**

In this study, we carried out a simulation study to evaluate four types of statistical approaches (fold-change/ratio, parametric tests/statistics, sensitivity index, and linear models) with different scenarios of RNAi screenings for drug sensitivity studies. With the simulated datasets, the linear model resulted in significantly lower false-negative and false-positive rates. Based on the results of the simulation study, we then make recommendations of statistical analysis methods for high-throughput RNAi screening data in different scenarios. We assessed promising methods using real data from a loss-of-function RNAi screen to identify hits that modulate paclitaxel sensitivity in breast cancer cells. High-confidence hits with specific inhibitors were further analyzed for their ability to inhibit breast cancer cell growth. Our analysis identified a number of gene targets with inhibitors known to enhance paclitaxel sensitivity, suggesting other genes identified may merit further investigation.

**Conclusions:**

RNAi screening can identify druggable targets and novel drug combinations that can sensitize cancer cells to chemotherapeutic drugs. However, applying an inappropriate statistical method or model to the RNAi screening data will result in decreased power to detect the true hits and increase false positive and false negative rates, leading researchers to draw incorrect conclusions. In this paper, we make recommendations to enable more objective selection of statistical analysis methods for high-throughput RNAi screening data.

## Background

Over the last decade, short RNA molecules (~20 to 30 nt) have emerged as critical regulators of the expression and function of eukaryotic genes. In particular, RNA interference (RNAi) is a valuable tool for modulating gene expression through the introduction of short interfering RNAs, including small interfering RNAs (siRNAs) and short hairpin RNAs (shRNAs) [1]. With its ability to silence genes in mammalian cells, RNAi has emerged as a powerful technology to knock down specific genes for functional analysis and for therapeutic purposes, particularly as we continue to learn more about specific genes involved in disease processes [2]. Recent research has focused on the use of high-throughput screens to analyze gene expression in cancer cell lines. Several RNAi studies conducted with human tumor cell lines, using synthetic siRNAs/shRNAs targeting defined gene families or genomic-wide libraries, have identified modulators of drug sensitivity [3-6].

Large-scale systematic RNAi screens aim to test hundreds, or even thousands, of siRNAs/shRNAs to identify hits rapidly and accurately. One major challenge of data processing and analysis for siRNA or shRNA screens in cancer research is to identify efficiently and accurately genes that, when lost, significantly reduce or increase cell growth/viability in response to chemical treatment. Two types of error can occur with screening experiments: false-positives and false-negatives. Strategies to reduce false-positives and false-negatives in the laboratory setting focus on making technical and procedural improvements and increasing the number of replicate measurements. It is also important to realize that enhanced statistical analysis methods also play an essential role in reducing error.

A number of statistical approaches have been applied to the analysis of high-throughput RNAi data. In their application, however, it is unclear whether: (1) effects of both the drug and the RNAi, as well as their interaction effect, are taken into consideration; (2) quantitative variation between and within replicates is taken into account in the estimation; and (3) decision error rates false-positive and false-negative are appropriately controlled. In this study, we carried out a simulation study to evaluate and compare statistical approaches for using RNAi screens to identify genes that alter sensitivity to chemotherapeutic drugs. We focused on combined RNAi and drug effect on cell viability, control of false-positive and false-negative rates, and the influence of drug concentration on the statistical power. The methods being evaluated were also applied to a real loss-of-function RNAi screening dataset to identify genes that modulate paclitaxel sensitivity in breast cancer cells.

## Methods

### Data processing and normalization

Several sources of noise, including technical and procedural factors, may influence measurement quality, generating inferential errors. Usually normalization is done prior to data analysis in RNAi screening studies such that variations contributed by unequal amounts of cells and/or RNAi are substantially reduced. Within-plate normalization can be performed using the non-silencing RNAi controls in the plate as a reference to give a relative measurement of target-gene knockdown effect, often adjusting for the variance by dividing by the standard deviation (SD) or median absolute deviation (MAD). Some approaches use a positive control or both positive and negative controls [7], others do not use a control, including Z score/robust Z score and B score [8]. Across-plate normalization is the process that makes measurements comparable across culture plates by removing systematic plate-to-plate variation. Common approaches include mean- or median-centered normalization, and standardization. In drug sensitivity studies, however, it is important to realize that such approaches can conceal true differences between drug-treated and untreated plates and consequently produce false-negatives. Instead, the raw viability data can be centered using the global mean or median of untreated non-silencing (NS) siRNA controls. Therefore, we recommend including untreated NS controls in all culture plates for drug sensitivity studies.

### Statistical approaches

One major purpose of RNAi screens is to identify genes that mediate the effects on cells of certain conditions, such as treatment with a chemotherapeutic drug or endocrine therapy. Such experiments explore the effect of gene knockdown in treated versus untreated wells, aiming to find meaningful associations between genes and the treatment. Different rules have been used to identify hits. A commonly used parametric approach is mean ± kSD under the assumption of normality [8,9] or its more robust version by replacing SD with MAD [10]. When distribution is skewed, a quartile-based method is an option [11]. Strictly standardized mean difference (SSMD) was first proposed to assess siRNA effect size, and modified later to balance false-negatives and false-positives in hit selection [12,13]. In high-throughput RNAi screens designed for drug sensitivity studies, available statistical approaches are much fewer; most commonly used by biologists include fold-change methods (sometimes combined with percent cell viability), parametric two-sample tests such as the *t*-test and Z-factor and their variants, and sensitivity index (SI).

Under the assumption that most features are not significant in high-dimensional data analysis, feature selection methods like Lasso (L_1 _penalty) and Elastic Nets (both L_1 _and L_2 _penalty) and their variants are often found useful and efficient in dimension reduction and feature selection. However, it may be difficult to adopt similar strategies to RNAi screening studies for drug sensitivity evaluation because, firstly, our main interest focuses on testing the gene-drug interactions, therefore in addition to siRNAs whose gene-drug interaction effect showed significance, the final model also needs to include drug effect regardless of its statistical significance. This however cannot be guaranteed by the automatic variable selection methods mentioned above. Secondly, when the number of features (*p*) is larger than the number of samples (*n*), lasso methods can select at most *n *features. This may be problematic for RNAi screening studies where *p *> >*n *is usual (in practice such experiments are usually designed with 3, 6, or 9 -replicate). Thirdly, lasso methods generate a number of most important features. Nevertheless, for gene function and drug discovery purposes in a high-throughput screening experiment, finding features with a small effect can be substantively important and a ranked list of candidate features, based on their significance, are often helpful.

Based on above considerations, we conducted a simulation study to evaluate the performance of commonly applied methods: fold change, *t *test, SI. We also fit a linear model (LM) of the probability of being a hit for each gene with an interaction term of drug and RNAi effect on cell viability. Each method is described as below.

#### Fold change/ratio

Fold change is the most intuitive approach used to represent the relative cell viability between two conditions, which usually is calculated as average cell viability (measured, for example, through an Alamar blue dye assay) over all wells in a condition divided by average viability over all wells in another condition. Because most genes knocked down in a siRNA screen do not have a significant effect on cell viability/growth in the background of the treatment, the log2 viability ratios between treated and untreated wells will be around zero for most genes. An arbitrary cut-off level, such as two- or three-fold change, is typically used to select hits for further experiments and analysis.

#### Parametric tests/statistics

Many biologists favor tests of two-group comparison for their easy calculation and interpretation. One widely used test is Student's two-sample *t *test. For each siRNA, a *t *statistic, *Ti*, is computed, and an siRNA is considered significant if |*Ti *| exceeds some threshold. The Z-factor and Z'-factor have been used for similar purposes; however, such analysis is usually based on the difference of the averaged readings over replicates between treated and untreated groups. These methods are more sophisticated than fold-change in the sense that they not only consider the average ratios between the groups but also incorporate information on the variation of the measurements and the number of replicates in the experiment.

#### Sensitivity index (SI)

The SI method was developed to measure the influence of siRNA-induced gene knockdown on drug sensitivity, by estimating the difference between the expected and observed combined effects of RNAi and drug on cell viability. Different from the methods discussed above, the SI method estimates both the influence of siRNA-induced gene knockdown on drug sensitivity and the individual drug and RNAi effects. The SI index can be calculated for each siRNA as SI = (Rc/Cc*Cd/Cc)-(Rd/Cc), where Rc is the average viability in drug-untreated wells transfected with active siRNA, Rd is the average viability in drug-treated wells with active siRNA, Cc is the average viability in drug-untreated wells with control siRNA, and Cd is the average viability in drug-treated wells with control siRNA. The SI value ranges from -1 to 1, with positive values indicating a sensitizing effect and negative values indicating an antagonizing effect. The SI method makes no assumption about the underlying probability distribution and therefore no *p*-values can be calculated.

#### Linear models with an siRNA-drug interaction effect

The SI method attempts to estimate combined RNA and drug effect. Nevertheless, one major disadvantage of the SI method is that it ignores the cross-plate variation of a particular siRNA, as the calculation of sensitivity ratio (Rd/Cd)/(Rc/Cc) involves only averaged reading levels over the replicate plates. Model-based methods are often used for feature selection in other types of high-throughput genomic data, including gene-expression microarray data and single nucleotide polymorphism (SNP) data. In our study, we used a simple linear model with an interaction term to assess RNAi effect, drug effect, and their combined effect. Assuming normal distribution, a full linear model *D*_2 _of cell viability (Y) for each siRNA *i *can be constructed based on the predictor variables: drug effect (*x*_1*i*_, yes/no), RNAi effect (*x*_2*i*_, yes/no), and their interaction term (*x*_1*i *_*x*_2*i*_).

(1) $$Y_{i}=\beta_{0}+\beta_{1}x_{1i}+\beta_{2}x_{2i}+\beta_{3}x_{1i}x_{2i}+\varepsilon_{i}$$

This model not only allows for estimating the gene-drug effect but also takes into consideration the variance among the replicates in its estimations. A test based on the difference between the deviance of the null model (model with no explanatory variables) *D*_0 _and the deviance of the fitted full model *D*_2 _may yield significant result when the drug effect is significant, even if the siRNA does not have any effect on cell viability. Therefore, we calculated the difference between the residual deviance of the fitted full model *D*_2 _and the deviance of the reduced model *D*_1 _including only drug effect (*x*_1_):

(2) $$Y_{i}=\beta_{0}+\beta_{1}x_{1i}+\varepsilon_{i}$$

This statistic, *D*_1 _-*D*_2_, follows a chi-square distribution with 2 degrees of freedom. The *p*-value based on this statistic reflects the combined effect of drug and RNAi as well as the RNAi effect alone of the given siRNA. The reason we did not include RNAi effect (*x*_2_) in *D*_1 _is that a significant RNAi effect alone without a significant interaction effect with drug treatment also provides vital information about the gene that is silenced, which can be very useful in identifying novel therapeutic targets for future studies.

### Simulation of datasets

We evaluated the methods using datasets simulated to represent different scenarios corresponding to a given combination of parameters of number of true hits, the amount of noise, the skewness of the data, the strength of chemotherapeutic drug effect, and the RNAi effect. We focused on combined RNAi and drug effect on cell viability, control of false-positive and false-negative rates, and the influence of drug concentration on the statistical power.

Data for ten 96-well plates with three, six, nine, or twelve replicates were simulated. For each scenario, 500 simulations were carried out. For each simulation, a number of true hits were drawn randomly from the distribution Uniform{10, 11, ..., 60} with an average of 35 out of 900 siRNA wells being truly sensitizing or antagonizing. The viability measurements of the samples transfected with non-hits were generated from N(*μ_NH_*, σ^2^), with *σ *at values of 0.2, 0.4, 0.6, or 0.8. The distribution of true hits is assumed to have a shifted mean relative to the distribution of non-hits: N(*μ*_NH*_C_*1*_, *σ^2^*) with *C_1 _*> 1 for a sensitizing effect and N(*μ*_NH*_C_*2*_, *σ^2^*) with *C_2 _*< 1 for an antagonizing effect (*C_1 _*= 7, *C_2 _*= 0.15; *C_1 _*= 5, *C_2 _*= 0.3; *C_1 _*= 2, *C_2 _*= 0.5; and *C_1 _*= 1.25, *C_2 _*= 0.8). The average cell viability in control wells is usually higher than that in siRNA-transfected wells. The parameter of the chemotherapeutic drug effect *D *was used to tune the strength of such effect. Specifically, the distribution of drug-treated samples (with active or control siRNA) has a shifted mean relative to untreated: *μ_trt _*= *μ*_*un*t_*D, *D *= 0.3, 0.6, and 0.8, where *μ*_*unt *_= *μ*_*NH *_, *μ*_NH*_C_1 _, *μ*_NH*_C_2 _, or *μ*_NH*_K as previously defined. In addition, parameter *K *is defined to be K = 1.05, 1.10, 1.15, or 1.2, such that control wells have a distribution with mean *μ_ctl _*= μ_rna_*K, where *μ_rna _*= *μ*_*NH *_or *μ*_NH*_D. Parameters *μ_NH_*, *σ*, *C_1_*, *C_2_*, *D*, and *K *were chosen such that the simulated data would resemble data with different distributions and properties, similar to those we have observed in real siRNA screening experiments. In particular, *C_1 _*and *C_2 _*were chosen such that the sensitizing and antagonizing effects would be equal in magnitude in order to have roughly same number of true hits simulated in both directions of the effect.

To evaluate the robustness of the methods for skewed data, gamma distributions were used instead of normal. The shape and scale parameters of gamma distributions were calculated by solving *μ = rλ and σ^2 ^= rλ^2 ^*based on previously used parameters of normal distributions. The skewness value ($2/\sqrt{r}$) is taken to be 0.5, 1, 1.5, or 2. Two situations were considered: when a strong drug effect is present (D = 0.3) and when a weak drug effect is present (D = 0.8).

### Criteria for the evaluation of statistical approaches

In practice, RNAi screening studies often involve a great deal of variation and noise in the raw data. Moreover, due to cost constraints, the number of replicates is often very limited. Hypothesis-testing under such conditions is, therefore, error-prone, with errors falling into two types: type I error (false-positive, FP) and type II error (false-negative, FN). To evaluate the performance of the methods, we calculated the false-positive rate (FPR) and the false-negative rate (FNR) of each method and in each scenario:

$$\text{FPR}=\text{FP}/\left(\text{FP}+\text{TN}\right)=\text{1}-Specificity,$$

$$\text{FNR }=\text{ FN}/\left(\text{TP}+\text{FN}\right)\;=\text{ 1}-Sensitivity.$$

The FPR corresponds to the portion of genes that, when silenced, have no influence on drug sensitivity among those identified as influential by the method. The FNR corresponds to the portion of genes influencing drug sensitivity among those claimed non-influential by the method.

### Real data analysis

Paclitaxel is a potent anti-microtubule agent used in the treatment of patients with locally advanced and metastatic breast cancer. Despite its wide use, paclitaxel-based chemotherapy results in full response in only a small portion of patients; many patients have an incomplete response or are resistant to treatment. We performed a loss-of-function RNAi screen to identify genes that modulate paclitaxel sensitivity [14]. We targeted a subset of genes (n = 428) frequently found to be "deregulated" in breast cancers and known to be associated with a targeted pharmacological agent (i.e., druggable), with the idea these could be analyzed in preclinical models for synergistic activity with paclitaxel. An shRNA screen was initially performed to identify druggable gene targets; we then validated the top high-confidence hits from the shRNA screen by designing two independent siRNAs for each gene, to be assayed in two representative breast cancer cell lines, MDA-MB-231 and MDA-MB-468. The two cell lines were reverse-transfected with siRNAs complexed with lipid reagent in each well of a 96-well plate for 48 h and subsequently split into six replicate plates. Following transfection of siRNAs, plates/cells then were treated for 24 h ± paclitaxel (i.e., 3-replicate plates +paclitaxel, 3-replicate plates -paclitaxel) and incubated for an additional 72 h to allow for changes in cell viability. To account for plate-to-plate variability and to control for the effects of siRNA transfection, data were normalized to non-silencing (NS) siRNA or shRNA controls, which do not target any human gene, for all plates. The full experiment (i.e., transfection, drug treatment, viability assay, and data normalization) was repeated, resulting in high reproducibility Pearson's correlation coefficients ~0.70-0.80.

## Results

### Simulation study

We report nine most representative scenarios simulated separately for each of the three, six, nine, and twelve-replicate datasets as described above. Because no critical value/threshold can be universally applied to all methods, results based on significance thresholds of different methods are not directly comparable. For the purpose of fair comparison, we selected the same number of hits from each method according to the true number of hits simulated in each dataset. We ranked all genes based on their significance assessed by each method and selected the top *n_TH _*hits (*n_TH _= *number of true hits), with half in each direction. FPRs and FNRs were then calculated from 500 simulations for each scenario at common target error control.

We compared the accuracy of the methods at different combinations of level of noise, drug effect, and RNAi effect. Table 1 lists simulation features and ranks the four methods based on their performances for identifying influential siRNAs in each scenario. In real data analysis, the degree of noise can be estimated from the coefficient of variation (CV = *σ*/*μ*) or variance to the mean ratio (VMR = *σ^2^*/*μ*) within the untreated data. Similarly, the effect of siRNA and the effect of the chemotherapeutic drug may be estimated from Rc/Cc and Cd/Cc (see the SI method section), respectively.

**Table 1:** Recommendation of analysis method based on simulation study

Number of replicates	Scenario	Noise	Drug effect	RNA effect	Performance ranking order*
3	1	low	strong	strong	LM, *t*, SI, FC
	2	moderate	strong	strong	LM, *t*, SI, FC
	3	strong	strong	strong	LM, *t*, SI, FC
	4	low	moderate	strong	LM, *t*, SI, FC
	5	low	weak	strong	LM, *t*, SI, FC
	6	moderate	weak	strong	LM, *t*, SI, FC
	7	strong	weak	strong	LM, SI, *t*, FC
	8	low	strong	moderate	LM, *t*, SI, FC
	9	low	strong	weak	LM, *t*, SI, FC
6	1	low	strong	strong	LM, *t*, SI, FC
	2	moderate	strong	strong	LM, *t*, SI, FC
	3	strong	strong	strong	LM, *t*, SI, FC
	4	low	moderate	strong	LM, *t*, SI, FC
	5	low	weak	strong	LM, *t*, SI, FC
	6	moderate	weak	strong	LM, *t*, SI, FC
	7	strong	weak	strong	LM, *t*, SI, FC
	8	low	strong	moderate	LM, *t*, SI, FC
	9	low	strong	weak	LM, *t*, SI, FC
9	1	low	strong	strong	LM, *t*, SI, FC
	2	moderate	strong	strong	LM, *t*, SI, FC
	3	strong	strong	strong	LM, SI, *t*, FC
	4	low	moderate	strong	LM, *t*, SI, FC
	5	low	weak	strong	LM, *t*, SI, FC
	6	moderate	weak	strong	LM, *t*, SI, FC
	7	strong	weak	strong	LM, *t*, SI, FC
	8	low	strong	moderate	LM, *t*, SI, FC
	9	low	strong	weak	LM, *t*, SI, FC
12	1	low	strong	strong	LM, *t*, SI, FC
	2	moderate	strong	strong	LM, SI, *t*, FC
	3	strong	strong	strong	LM, SI, *t*, FC
	4	low	moderate	strong	LM, *t*, SI, FC
	5	low	weak	strong	LM, *t*, SI, FC
	6	moderate	weak	strong	LM, *t*, SI, FC
	7	strong	weak	strong	LM, *t*, SI, FC
	8	low	strong	moderate	LM, *t*, SI, FC
	9	low	strong	weak	LM, *t*, SI, FC

* The ranking is based on average FNRs and FPRs of the methods: linear model (LM), *t*-test (*t*), sensitivity index (SI), and fold-change (FC).

**Figure 1:**
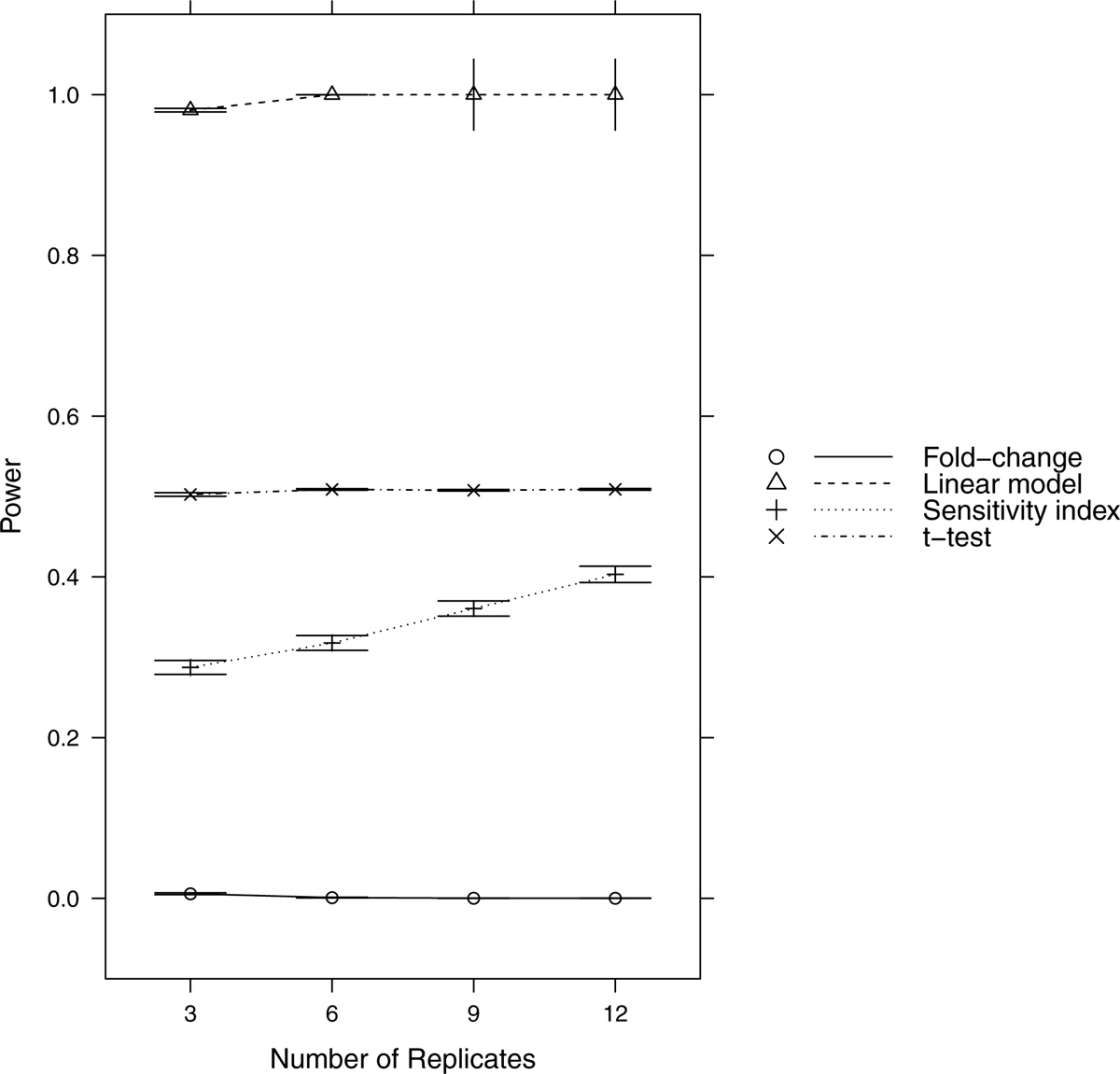
**Scenario 1**. Low noise, high drug effect, high RNA effect.

**Figure 2:**
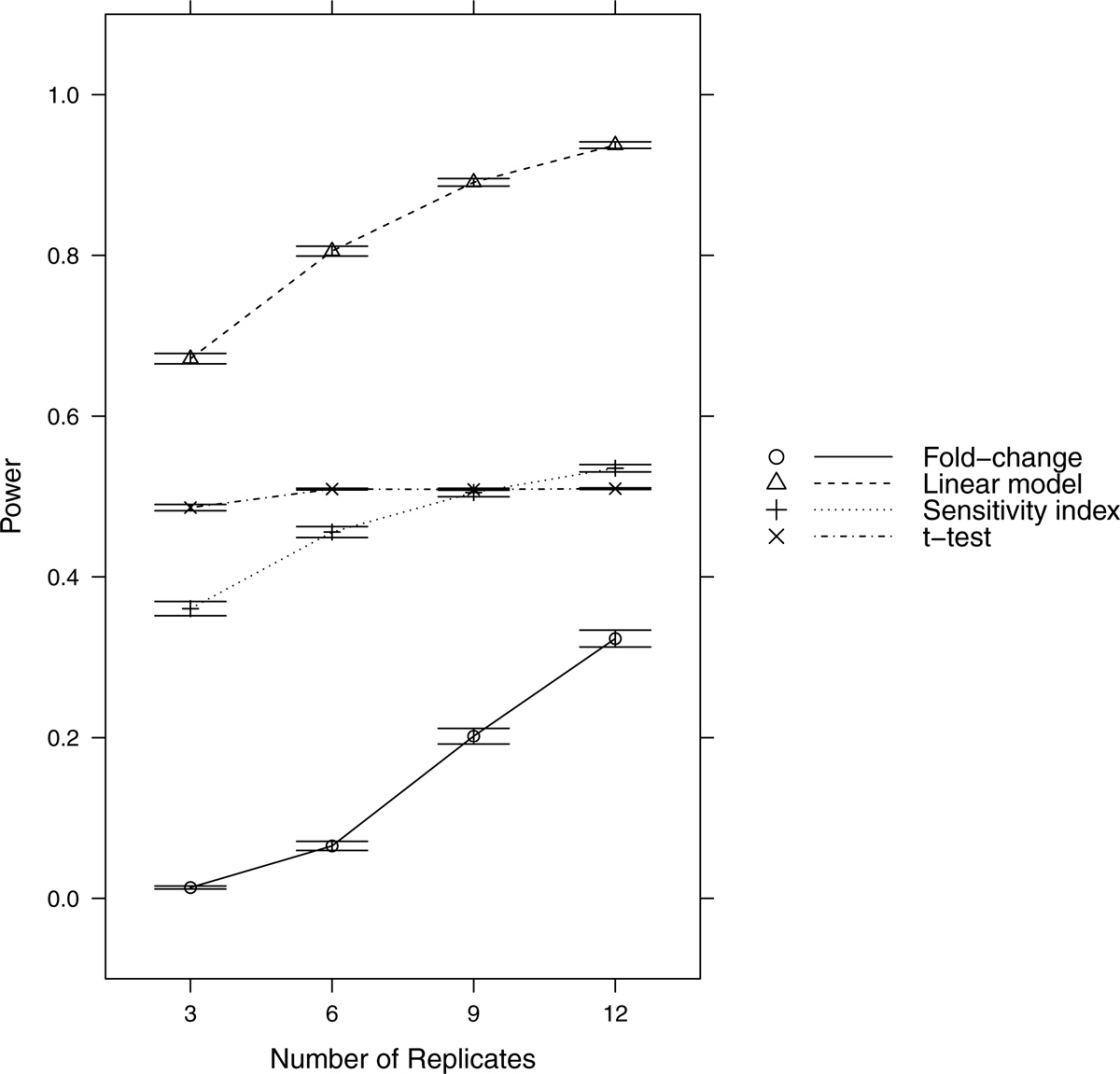
**Scenario 2**. Moderate noise, high drug effect, high RNA effect.

**Figure 3:**
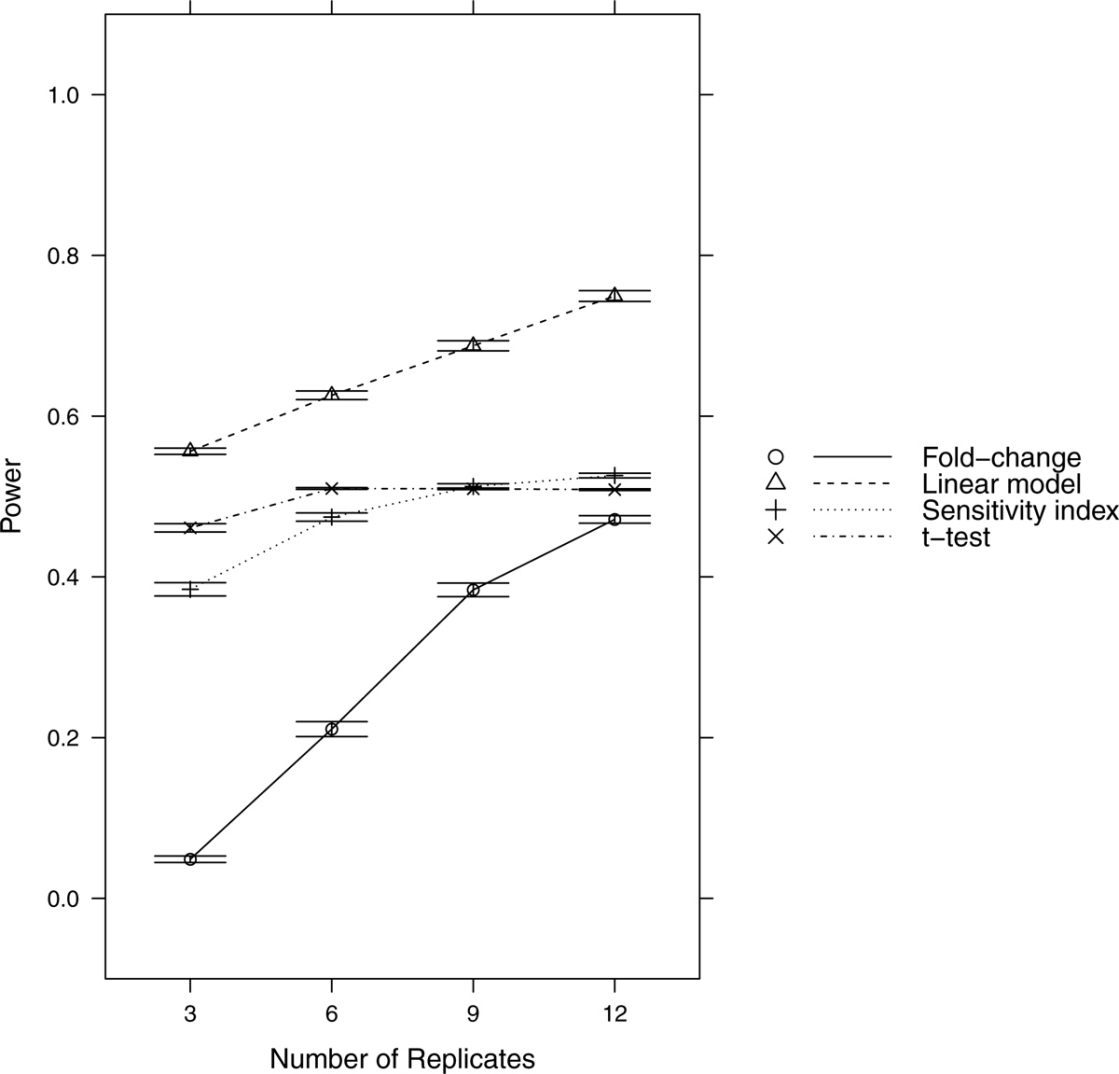
**Scenario 3**. High noise, high drug effect, high RNA effect.

**Figure 4:**
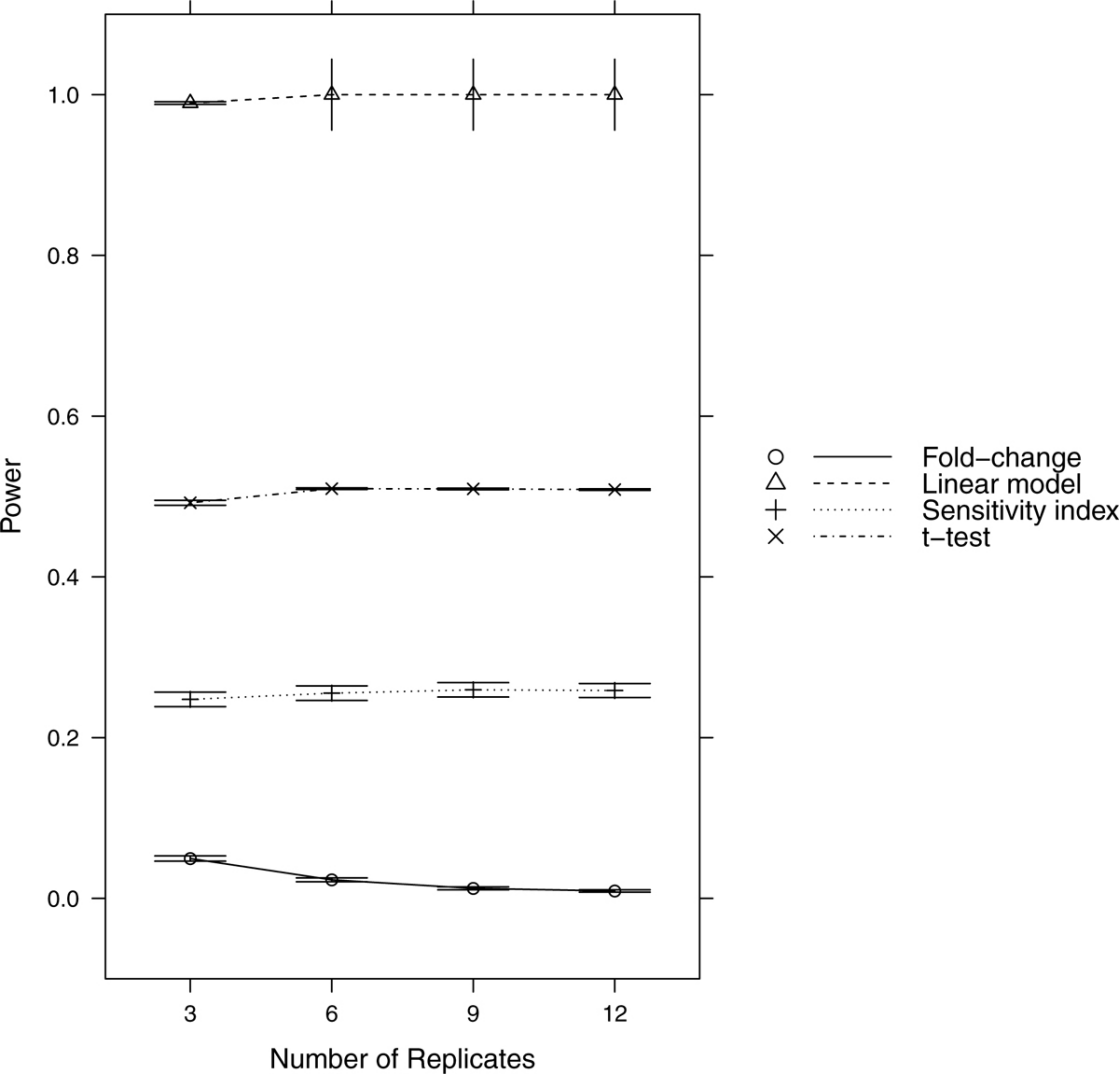
**Scenario 4**. Low noise, moderate drug effect, high RNA effect.

**Figure 5:**
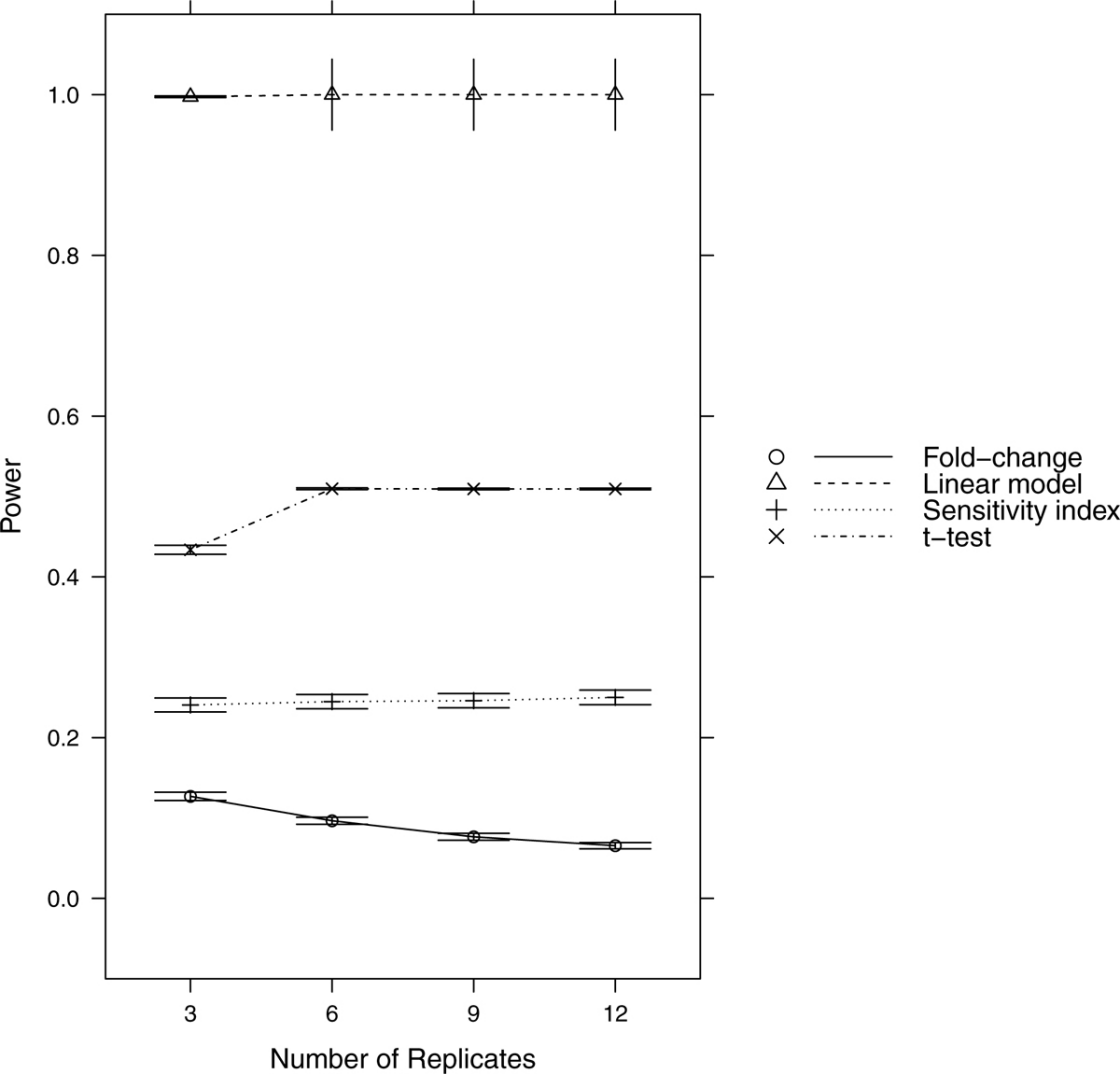
**Scenario 5**. Low noise, weak drug effect, high RNA effect.

**Figure 6:**
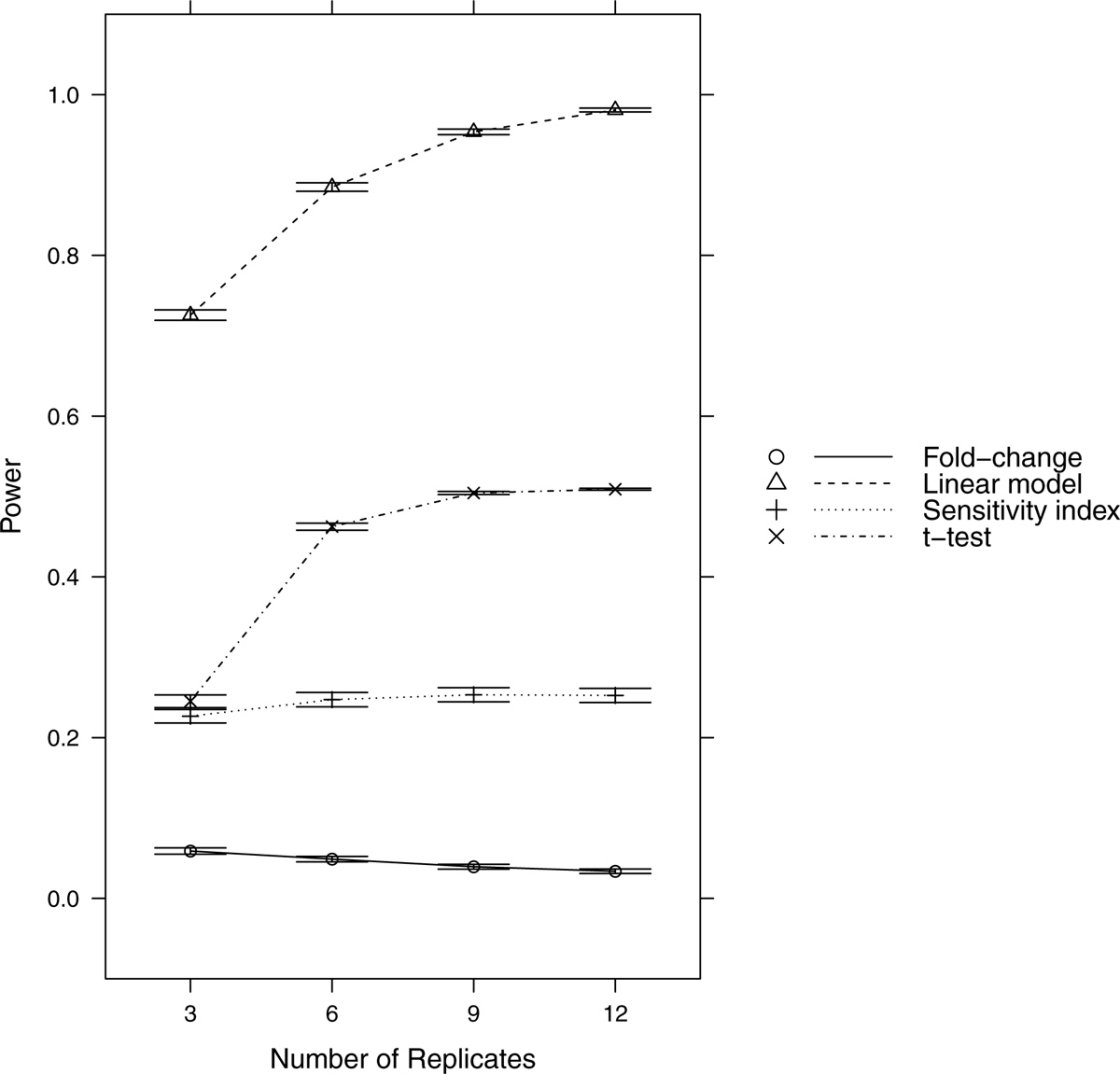
**Scenario 6**. Moderate noise, weak drug effect, high RNA effect.

**Figure 7:**
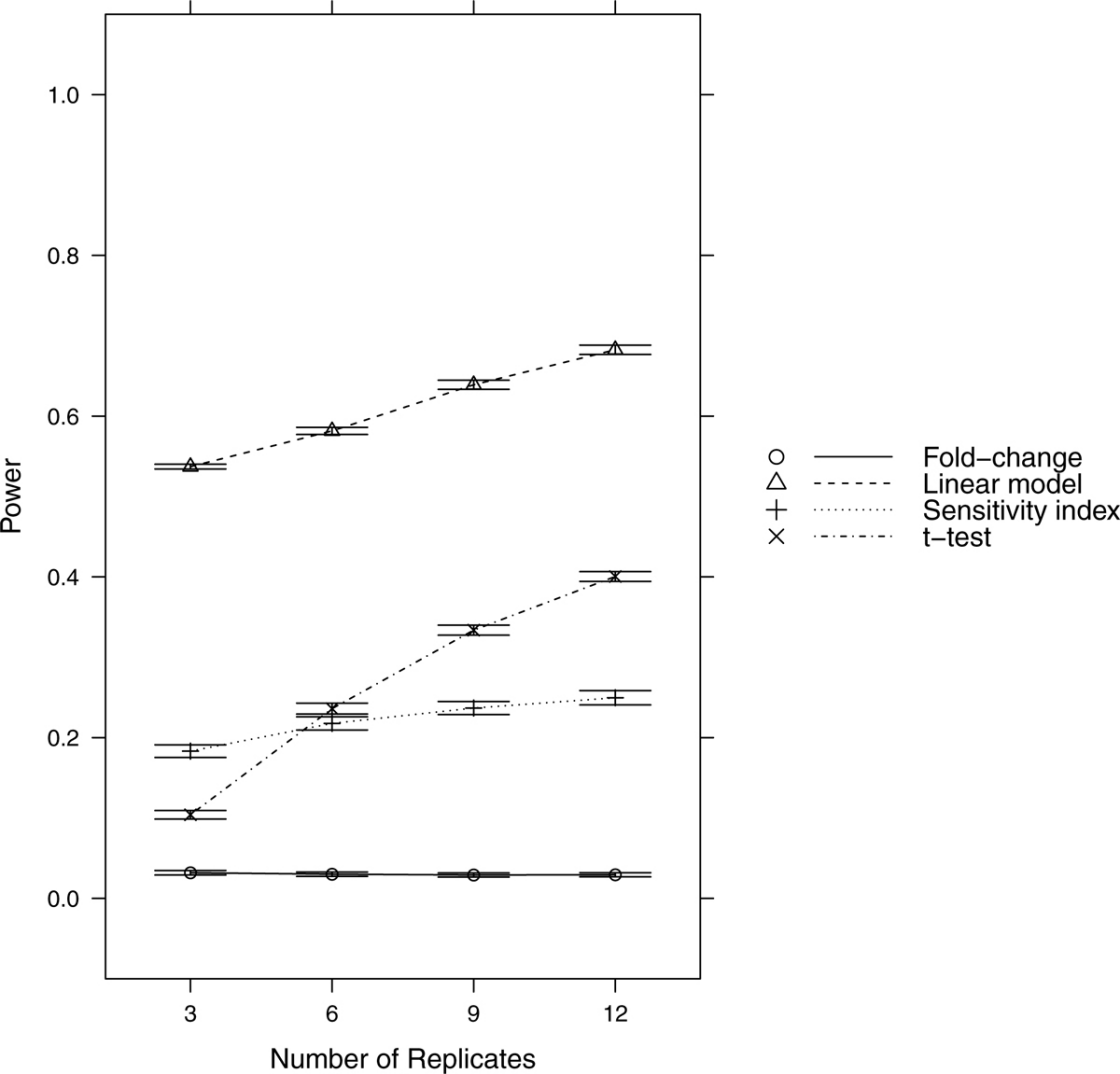
**Scenario 7**. High noise, weak drug effect, high RNA effect.

**Figure 8:**
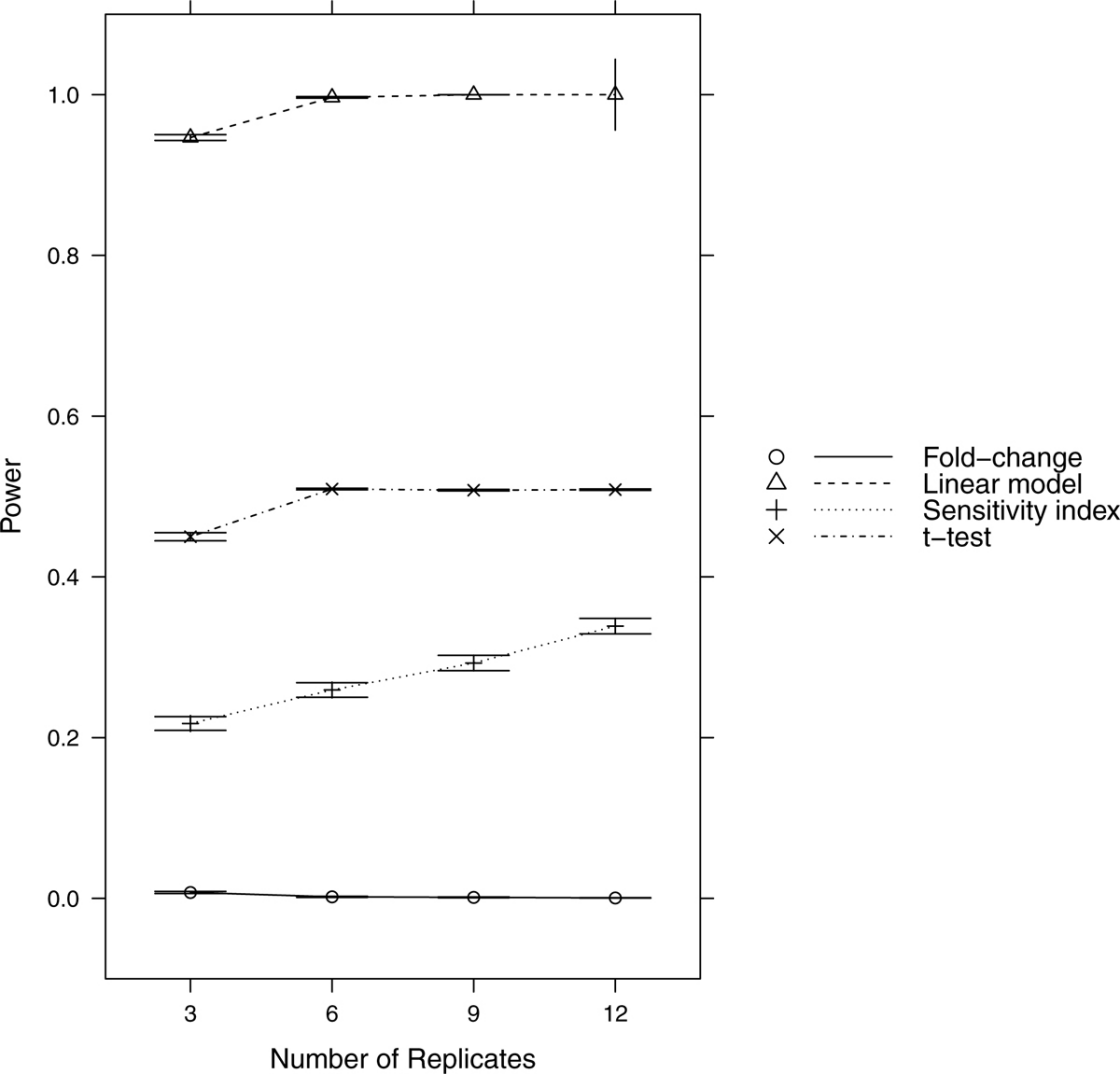
**Scenario 8**. Low noise, high drug effect, moderate RNA effect.

**Figure 9:**
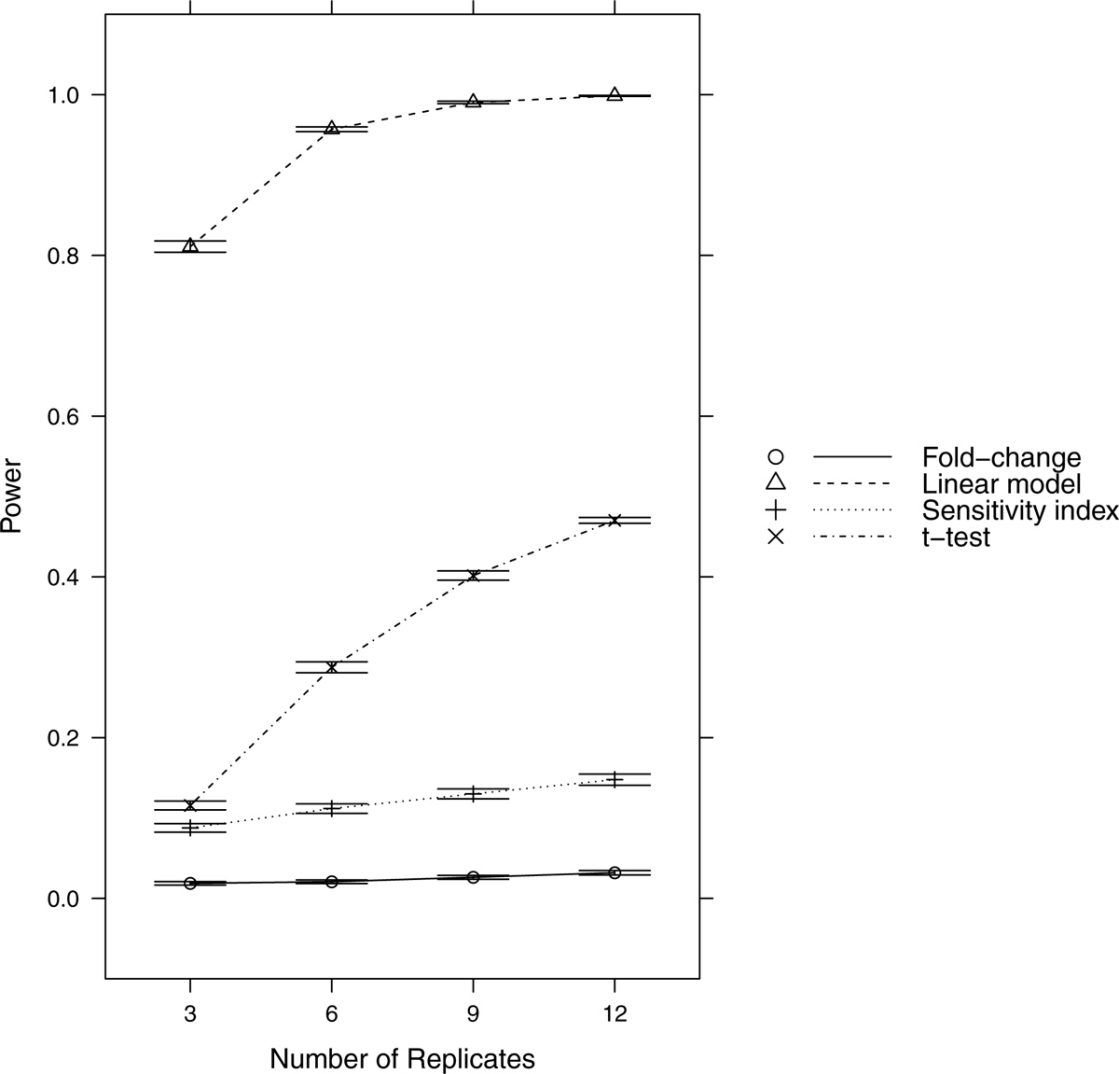
**Scenario 9**. Low noise, high drug effect, weak RNA effect.

Since power = sensitivity = 1-FNR, controlling FNR automatically controls power/sensitivity. Figures 1, 2, 3, 4, 5, 6, 7, 8, 9 show that the LM always has the lowest FNR among all four methods compared. The FNR decreases with the number of replicates in all scenarios for the *t *test, LM, and SI methods. In addition, the advantage of using the LM is obvious when the drug effect is low to moderate (in practice, such a drug effect can result from a low drug concentration). In such cases, the FNR of LM can be as low as < 10%, while other methods have a FNR higher than 40%.

When a strong drug effect is present, the SI method is less powerful with skewed data than with unskewed data. The LM method is very stable: FNR with skewed data is only slightly higher than with unskewed data in simulations with a small number of replicates. The *t*-test has a similar behaviour as the LM in this situation. On the other hand, when drug effect is weak, FNR of the LM decreases faster with number of replicates in the case of skewed data than in the case of unskewed data.

Our simulation study suggests that the LM method performs overwhelmingly better than all other methods considered. When the data have a strong drug and RNAi effect but with a small number of replicates, the *t*-test in general has a better performance than the SI and fold-change. However, a single *t*-test observation of cell viability from one experiment may not yield reliable results for a specific siRNA, because perceived variability in that siRNA when the target gene is knocked down may actually arise from experimental noise. The SI method may provide a useful alternative to the *t*-test, potentially resulting in a lower FPR/FNR when the data has a moderate to high level of noise but strong drug and siRNA effects. The fold-change method, on the other hand, is only suitable for data with few or no replicates, where hypothesis testing cannot apply.

#### Hits from shRNA/siRNA screening

After normalization, the SI method was initially applied to identify gene hits, as this method had recently been proposed and published as an approach to account for RNAi-drug interaction. The SI score was calculated for each of the shRNAs and siRNAs. Genes were then ranked according to the SI score, and the top hits for each cell line were selected for further analysis. After the simulation study described above was completed, we subsequently applied the t-test, fold-change, and LM methods to the same data. The top hits selected by SI also ranked very high on the list generated by LM, though a small number of mismatches were observed (for example, SP1 was identified as a top hit only in the MDA-MB-468 cell line with the SI method, but ranked high in both cell lines with the LM method). This is expected because the data has strong drug and RNAi effects; also we only validated top hits with the strongest combined effect.

FRAP1 (mTOR) was a hit in both cell lines, as anticipated. This gene is a known target for enhancing paclitaxel sensitivity and was used as a positive control in each plate of our screen to allow for cross-plate comparisons of drug sensitivity [5]. EGFR was a top hit in MDA-MB-468 cells, a breast cancer cell line that overexpresses EGFR and that is resistant to erlotinib (an EGFR inhibitor); erlotinib previously has been shown to enhance paclitaxel sensitivity [15,16]. Centromere protein F, CENPF, a hit in both cell lines, is associated with the centromere-kinetochore complex and may play a role in chromosome segregation during cell mitosis. CENPF is a target of farnesyltransferase inhibitors (FTIs), known to act synergistically to inhibit cell growth in combination with agents that prevent microtubule depolymerization, such as paclitaxel [17].

PPM1D, SP1, and TGF-β1 were hits of particular interest, as these genes encode proteins with known chemical inhibitors, which could be tested in combination with paclitaxel for biological effect. When the chemical inhibitors CCT007093 (PPM1D inhibitor) and mithramycin (SP1-binding inhibitor) were used in combination with paclitaxel, we observed synergistic growth inhibition of breast cancer cell cultures. We observed similar results with the transforming growth factor beta receptor inhibitor, LY2109761, which targets the TGF-β1 signaling pathway; LY2109761 plus paclitaxel synergistically inhbited growth of breast cancer cell lines in 3D culture. These examples provide strong evidence that validate our identified druggable gene targets that modulate paclitaxel sensitivity.

## Discussion

It is typical that high-throughput RNAi screening studies attempt to test hundreds or thousands of siRNAs with a relatively small number of replicates for each. It has been brought to our attention that popular statistical approaches for analyzing such data, although commonly applied, appear to have drawbacks in terms of efficiency and accuracy. Using simulated datasets for different scenarios, we evaluated and compared these approaches to the linear model we conducted for estimating both the influence of siRNA-induced gene knockdown on drug sensitivity and the individual effects of the drug and siRNA on cell viability. Overall, the LM method outperforms other evaluated methods mainly because it not only takes the variation among replicate measurements but also focuses on estimating the combined effect of drug and RNAi by incorporating an interaction term in the model.

Because a relatively small number of true hits (10 to 60) in our study were simulated among 900 siRNAs, the numbers of both false-negatives and true-positives are small relative to the total number of siRNAs, making the FNR very sensitive to the total number of true-positives. On the other hand, the number of true-negatives is large relative to the number of false-positives; therefore FPRs are, in general, very low (< 5%).

The *t *test and the LM method we applied require a normality assumption for the residuals, which may not hold in real data analysis. Therefore we have also considered non-parametric tests such as the Wilcoxon rank-sum test. Nevertheless, because non-parametric tests trade-off power for increased robustness and wider applicability, the sample size for most RNAi screening studies will be too small to enable conclusions from non-parametric tests with the same degree of confidence as from parametric tests. Intuitively, a mixed effect model can be used to take possible correlation between controls and siRNAs on the same plate into consideration. With the very small sample size typical of RNAi screening studies, however, a mixed effects model would lack sufficient power because of the degrees of freedom added to the model due to a nested factor. A practical solution is to apply normalization techniques prior to the statistical analysis to minimize between-plate variation.

As previously mentioned, in practice, low drug effect usually results from low drug concentration. Interestingly, recent studies have identified targets that sensitize cancer cells to chemotherapy drugs of a much lower concentration than otherwise required, such as paclitaxel for non-small-cell lung cancer cells [18]. In such studies, analysis based on the LM can be much more powerful and more accurate than the other methods discussed, especially the ratio-based approaches.

## Conclusions

RNAi screening can identify genes that mediate sensitivity or resistance to certain chemotherapeutic drugs and novel drug combinations that can sensitize cancer cells to a chemotherapeutic drug. However, applying an inappropriate statistical method or model to RNAi screening data will result in decreased power to detect true hits, increase the false-positive and false-negative rates, and consequently lead to incorrect conclusions. Based on the results of our simulation study, the authors have made recommendations to enable objective selection of statistical analysis methods for high-throughput RNAi screening data.

## Competing interests

The authors declare that they have no competing interests.

## Authors' contributions

FY performed the simulations, applied the various methodologies for analyzing RNAi screening data, and performed the statistical analyses to compare these methodologies. JAB performed the experiments to generate the real-world breast cancer datasets, and JAP supervised this laboratory research. YS guided and supervised the overall project. All authors contributed to writing of the manuscript.
